# Effects of ultrafine particles on the allergic inflammation in the lung of asthmatics: results of a double-blinded randomized cross-over clinical pilot study

**DOI:** 10.1186/s12989-014-0039-3

**Published:** 2014-09-10

**Authors:** Frank Schaumann, Cornelia Frömke, Dorothea Dijkstra, Francesca Alessandrini, Horst Windt, Erwin Karg, Meike Müller, Carla Winkler, Armin Braun, Armin Koch, Jens Michael Hohlfeld, Heidrun Behrendt, Otmar Schmid, Wolfgang Koch, Holger Schulz, Norbert Krug

**Affiliations:** 1 Fraunhofer Institute for Toxicology and Experimental Medicine, Nikolai-Fuchs-Str. 1a, Hannover, 30625, Germany; 2 Hannover Medical School, Hannover, Germany; 3 Center of Allergy and Environment (ZAUM), Technische Universität and Helmholtz Zentrum München, Member of the German Center for Lung research (DZL), Munich, Germany, Munich, Germany; 4 Cooperationgroup Comprehensive Molecular Analytics (CMA), Joint Mass Spectrometry Centre (JMSC), Helmholtz Zentrum München, Munich, Germany; 5 Comprehensive Pneumology Center, Institute of Lung Biology and Disease, Helmholtz Zentrum München, Member of the German Center for Lung Research, Munich, Germany; 6 Institute of Epidemiology I, Helmholtz Zentrum München, Munich, Germany; 7 Biomedical Research in Endstage and Obstructive Lung Disease Hannover (BREATH), Member of the German Center for Lung Research, Hannover, Germany

**Keywords:** Ultrafine particles, Asthma, Pulmonary inflammation, Aerosol exposure, Aeroallergen

## Abstract

**Background:**

Epidemiological and experimental studies suggest that exposure to ultrafine particles (UFP) might aggravate the allergic inflammation of the lung in asthmatics.

**Methods:**

We exposed 12 allergic asthmatics in two subgroups in a double-blinded randomized cross-over design, first to freshly generated ultrafine carbon particles (64 μg/m^3^; 6.1 ± 0.4 × 10^5^ particles/cm^3^ for 2 h) and then to filtered air or vice versa with a 28-day recovery period in-between. Eighteen hours after each exposure, grass pollen was instilled into a lung lobe via bronchoscopy. Another 24 hours later, inflammatory cells were collected by means of bronchoalveolar lavage (BAL). (Trial registration: NCT00527462)

**Results:**

For the entire study group, inhalation of UFP by itself had no significant effect on the allergen induced inflammatory response measured with total cell count as compared to exposure with filtered air (p = 0.188). However, the subgroup of subjects, which inhaled UFP during the first exposure, exhibited a significant increase in total BAL cells (p = 0.021), eosinophils (p = 0.031) and monocytes (p = 0.013) after filtered air exposure and subsequent allergen challenge 28 days later. Additionally, the potential of BAL cells to generate oxidant radicals was significantly elevated at that time point. The subgroup that was exposed first to filtered air and 28 days later to UFP did not reveal differences between sessions.

**Conclusions:**

Our data demonstrate that pre-allergen exposure to UFP had no acute effect on the allergic inflammation. However, the subgroup analysis lead to the speculation that inhaled UFP particles might have a long-term effect on the inflammatory course in asthmatic patients. This should be reconfirmed in further studies with an appropriate study design and sufficient number of subjects.

## 1 Background

Epidemiological studies have shown an association between increased ambient particle concentrations and adverse respiratory and cardiovascular health effects [1]-[3]. Ultrafine particles (UFP) as a component of ambient particles, with an aerodynamic diameter < 0.1 μm may contribute to these health effects [4]-[7]. UFP are characterized by a high number and a low mass concentration in the ambient air. They provide a large surface area per mass for interaction with biological structures and molecules [8]. Compared to larger particles, they have a higher deposition rate in the peripheral lung and an enhanced capability to produce reactive oxygen species [8]-[11].

Regarding asthma, a disease characterized by airflow limitation due to chronic airway inflammation, there is a clear association of particulate air pollution with increasing exacerbations and hospital admissions [12]-[15]. A study on subjects with asthma revealed that the concentration of UFP correlated closely with alterations in lung function [16]. McCreanor et al. [17] showed in a cross-over study that diesel particles, as the major source of urban UFP, alter the lung function of asthmatic patients. These studies suggest that especially patients with allergic asthma are more susceptible to the effect of ultrafine particle exposure [7],[18],[19].

Several animal studies have analyzed the effect of UFP on allergic sensitization. In particular, carbon black, which resembles the carbonaceous core of diesel exhaust, enhanced the sensitization towards a harmless antigen in several studies [20]-[22]. Alessandrini et al. demonstrated that the exposure to ultrafine carbon particles prior to allergen challenge exerts strong adjuvant effects on the allergic airway inflammation [23].

Controlled clinical exposure studies with carbon UFP have demonstrated a high pulmonary deposition in healthy subjects [24], which was further increased in subjects with asthma [24],[25]. These studies demonstrated altered peripheral blood leukocytes distribution and expression of adhesion molecules. However, short term effects on inflammatory cell counts in induced sputum were not observed [25],[26]. While these studies included only mild and stable asthmatics, controlled clinical studies evaluating the effects of UFP on exacerbated allergic airway inflammation are lacking so far.

Therefore, the aim of this study was to test the hypothesis that pre-exposure to UFP may aggravate an induced allergic airway inflammation in asthmatic patients. The study design consisted of a controlled inhalation of ultrafine carbon particles or filtered air for two hours which was followed by a segmental allergen challenge 18 hours later in mild asthmatics [27]. The particle effect on the allergic inflammation was compared in a randomized, double-blind crossover design: Allergic asthmatics were randomly assigned to two exposure subgroups starting with either ultrafine carbon particles followed by filtered air (sequence A), or filtered air followed by ultrafine carbon particles (sequence B). The consecutive inhalative exposures were separated by a recovery period of at least 28 days.

## 2 Results

### 2.1 Study subjects

**Figure 1:**
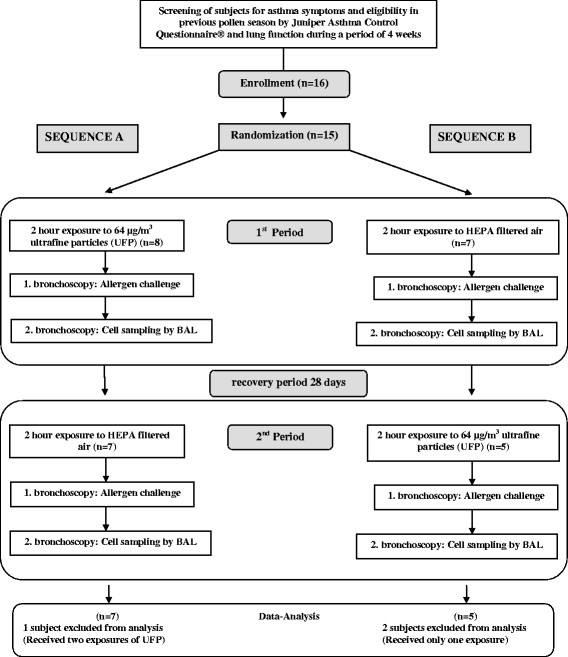
Flowchart of the study design.

Sixteen subjects with allergic asthma were enrolled in this study of which 15 were randomized (Figure 1). Finally, only 12 of the randomized subjects (4 women and 8 men in the age of 25–46 years) were included in the data analysis, since two subjects received only one exposure for personal reasons or illness and one subject was inadvertently exposed to UFP in both periods. Characteristic of study subjects are shown in Table 1.

**Table 1:** Characteristic of study subjects

**Characteristic of study subjects**	**N = 12**
Female sex − no. (%)	4 (33.3)
Age − years	38 [34.2;41.5]
Body weight - BMI	25.8 [23.8;27.8]
FEV_1_ − % predicted value	96.4 [88.8;103.9]
Methacholine PC_20_ − mg/ml	1.3 [0.3;5.3]
IgE − IU/ml	423.8 [79.0; 768.5]
Segmental allergen dose − SQE	541.8 [237.2;846.3]

Absolute and relative frequencies of women, mean of study subjects and corresponding 95%-confidence interval for all other baseline characteristics. Confidence interval for metacholine concentration is computed with logarithmic transformation; BMI: Body mass index, FEV1: forced expiratory volume in 1 second, Methacholine PC20:metacholine concentration to induce a 20% decrease in FEV_1_, IgE: total serum IgE (immunonglobulin E), SQE: standard quality unit.

**Figure 2:**
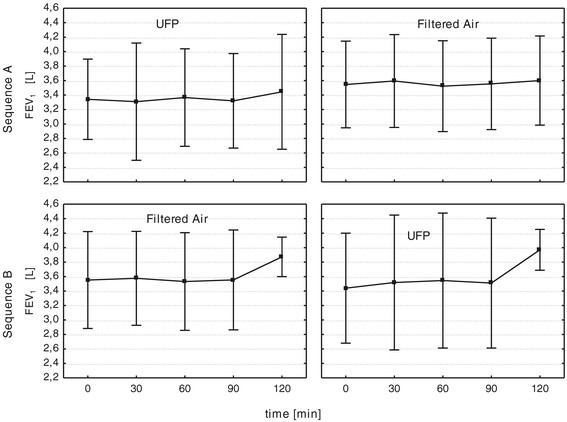
**FEV**_**1**_**prior and during exposure with UFP and filtered air for exposure sequence A and B.**

No serious adverse events occurred during the study. We found no significant changes in lung functions parameters from the baseline during the exposure to UFP or filtered air, respectively (Figure 2). Furthermore, we observed no accompanying changes in oxygen saturation during exposures as measured by pulse oximetry (data not shown).

### 2.2 Exposure data

During the exposures with UFP the mean particle mass and number concentration was 64.3 ± 11.2 μg/m^3^ with a mean number concentration of 6.1 ± 0.4 × 10^5^ particles/cm^3,^ respectively. The median diameter of the UFP was 49.9 ± 2.0 nm with a mean geometric standard deviation during the exposures of 1.62. During the control exposures with filtered air the mean number concentration was 89.4 particles/cm^3^ and the mass concentration was below detection limit. During all exposures the relative humidity and temperature were in a range of 41–50% and 22–23°C, respectively.

### 2.3 Exposure effects on the allergic airway inflammation

To investigate the effect of the exposures on the allergic inflammation we determined the absolute numbers of different cell populations and the concentration of mediators in the BAL fluid after segmental challenge of the lungs with grass pollen extract compared to the control challenge with saline.

**Figure 3:**
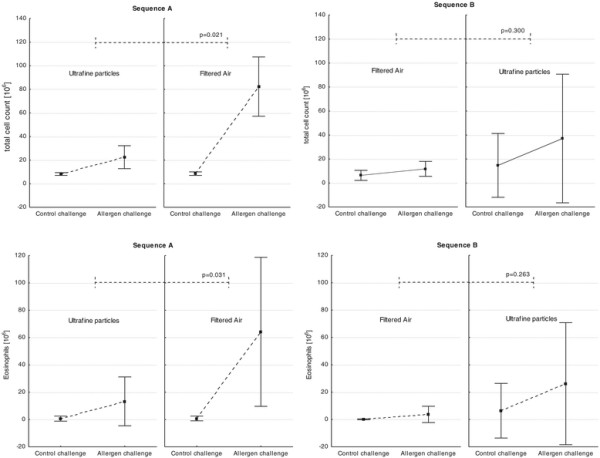
**Total cells and absolute eosinophils in BAL in the sequence (A): 1**^**st**^**Period: UFP – 2**^**nd**^**Period: Filtered air and sequence (B): 1**^**st**^**Period: Filtered air – 2**^**nd**^**Period: UFP.** Mean ± SD.

**Figure 4:**
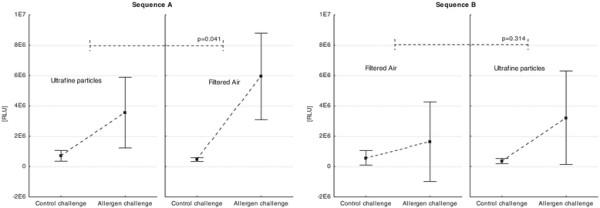
**Treatment differences between UFP and filtered air in sequence (A): 1**^**st**^**Period: UFP – 2**^**nd**^**Period: Filtered air and sequence (B): 1**^**st**^**Period: Filtered air – 2**^**nd**^**Period: UFP on oxidant radical generation of BAL cells after PMA stimulation measured via chemiluminescence (RLU: relative luminescence units).** Mean ± SD.

For the primary statistical analysis of the cross-over-design, we used an analysis of variance model with mixed effects including the fixed effect of the exposures, the periods and sequences and the random effect of the patients within sequences to model the total cell numbers. When all data were combined this global analysis showed no significant treatment differences between the exposures with UFP and filtered air on the primary endpoint (total cell numbers; p = 0.188) and the key secondary endpoint (absolute eosinophil numbers; p = 0.21) in the BAL fluid after the allergen challenge (Table 2). However, we identified sequence dependent differences in the allergic inflammation between the exposures of UFP and filtered air. We observed a more pronounced allergic inflammation after exposure with UFP in the first and filtered air in the second period (sequence A) compared to the exposure to UFP in the first period for the primary endpoint (total cells; p = 0.021) and the key secondary endpoint (absolute eosinophil numbers; (p = 0.031) as well as for monocytes (p = 0.013) (Figure 3, Table 3). Additionally, the potential of BAL cells to generate oxygen radicals after stimulation of PMA was significantly increased under filtered air in the second period compared to UFP (Figure 4, p = 0.041). Furthermore, the cytokines/chemokines IL-6, MCP-1, and TNF-α were significantly elevated at the same time point (Table 4, p < 0.05).

**Table 2:** Main effect analysis of primary endpoints of the global study design (including all subjects and exposures of sequence A and B)

***Global mixed model main effect analysis of the cross-over design***					
**BAL cells [10**^**6**^**]**	**UFP**_**mean (allergen-saline)**_	**Filtered air (FA)**_**mean (allergen-saline)**_	**Treatment effect (UFP-FA)**	**95%-confidence interval**	***p*-value**
Total cells	20.1	45.7	−25.6	−64.4; 13.2	0.188
Eosinophils	18.6	38.9	−20.3	−52.9; 12.2	0.212

Main effect analysis analyzing total BAL cells and eosinophils using a mixed model with fixed factors exposure, period, sequence, and subjects within sequence as random factor. Estimated mean differences (between allergen challenge and saline) of total cells and absolute eosinophils in BAL are shown 42 h after ultrafine particle (UFP) and filtered air (FA) exposure. Additionally, absolute treatment differences between UFP and filtered air, with 95% confidence levels and p-values are shown.

**Table 3:** Post hoc sequence analysis of total and differential cell count in BAL

***Sequence (A): 1***^***st***^ ***Period: UFP – 2***^***nd***^ ***Period: Filtered air***					
BAL Cells [10^6^]	UFP _mean (allergen-saline)_	Filtered air _mean (allergen-saline)_	Treatment effect (UFP-FA)	95%-confidence interval	*p*-value
Total cells	13.4	75.1	−61.6	−110.4; −12.8	0.021
Eosinophils	13.2	64.1	−50.9	−95.5; −6.3	0.031
Monocytes	0.9	5.7	−4.8	−8.3; −1.4	0.013
Macrophages	0.3	6.9	−6.6	−18.7; 5.5	0.232
Neutrophils	−0.8	1.9	−2.7	−7.6; 2.1	0.219
Lymphocytes	0.0	1.0	−1.0	−3.1; 1.2	0.313
***Sequence (B): 1***^***st***^ ***Period: Filtered air – 2***^***nd***^ ***Period: UFP***					
BAL Cells [10^6^]	Filtered air _mean (allergen-saline)_	UFP _mean (allergen-saline)_	Treatment effect (UFP-FA)	95%-confidence interval	*p*-value
Total cells	4.5	29.3	24.8	−33.2; 82.8	0.300
Eosinophils	3.7	26.1	22.5	−25.5; 70.4	0.263
Monocytes	0.4	3.5	3.2	−5.7; 12.0	0.377
Macrophages	0.6	0.7	0.1	−5.8; 6.0	0.951
Neutrophils	0.2	1.1	0.8	−2.2; 3.8	0.492
Lymphocytes	−0.1	0.6	0.6	−1.9; 3.2	0.531

**Table 4:** Post hoc sequence analysis of mediators (cytokines and chemokines) in BAL fluid

***Sequence(A): 1.Period: UPF – 2.Period: Filtered air***					
Mediators BAL [pg/ml]	UFP _mean (allergen-saline)_	Filtered air (FA) _mean (allergen-saline)_	Treatment effect (UFP-FA)	95%-confidence interval	*p*-value
IL-6	−3.0	55.0	−58.0	−102.8; −13.1	0.019
MCP-1	38.0	210.0	−171.9	−303.3; −40.6	0.019
TNF-α	0.2	2.1	−1.9	−2.8; −1.0	0.002
Eotaxin	2.3	10.7	−8.4	−18.9; 2.1	0.097
IL10	0.9	7.2	−6.4	−14.3; 1.6	0.099
CD40L	2.4	2.3	0.2	−5.8; 6.1	0.948
GM-CSF	5.5	6.1	−0.6	−5.5; 4.3	0.779
IFN-γ	−0.5	0.1	−0.6	−2.5; 1.4	0.527
IL-13	8.8	67.1	−58.3	−137.5; 20.9	0.122
IL-17	0.1	0.3	−0.2	−0.8; 0.4	0.477
IL-2	1.1	1.00	0.1	−3.0; 3.2	0.928
IL-4	1.3	13.0	−11.8	−30.9; 7.4	0.184
IL-5	128.6	73.3	55.4	−313.1; 423.8	0.726
IL-7	−2.0	−0.4	−1.6	−19.0; 15.7	0.827
IL-8	20.9	48.0	−27.1	−96.1; 42.0	0.375
IL-1β	−0.1	1.0	−1.1	−2.9; 0.7	0.174
IL12p70	−0.3	0.01	−0.3	−1.0; 0.4	0.356
***Sequence(B): 1.Period: Filtered air – 2.Period: UFP***					
Mediators BAL [pg/ml]	Filtered air (FA) _mean (allergen-saline)_	UFP _mean (allergen-saline)_	Treatment effect (UFP-FA)	95%-confidence interval	*p*-value
IL-6	12.3	30.5	18.2	−19.4; 55.9	0.250
MCP-1	27.4	113.5	86.1	−98.7; 270.9	0.265
TNF-α	0.2	1.3	1.1	−1.4; 3.5	0.289
Eotaxin	0.6	5.7	5. 2	−4.6; 14.9	0.216
IL10	0.9	8.5	7.7	−5.4; 20.8	0.179
CD40L	0.0	0.1	0.1	−0.2; 0.5	0.374
GM-CSF	2.4	2.5	0.1	−1.7; 1.9	0.910
IFN-γ	−0.3	0.4	0.6	−0.5; 1.7	0.181
IL-13	3.0	63.5	60.4	−46.7; 167.6	0.192
IL-17	0.0	1.2	1.2	−1.1; 3.4	0.237
IL-2	1.1	4.3	3.1	−9.9; 16.2	0.540
IL-4	0.0	7.8	7.8	−5.5; 21.0	0.178
IL-5	29.0	75.2	46.2	−39.1; 131.4	0.208
IL-7	−1.0	−0.8	0.3	−7.2; 7.8	0.928
IL-8	13.1	21.5	8.4	−28.9; 45.8	0.565
IL-1β	−0.1	0.1	0.1	−0.2; 0.5	0.374
IL12p70	−0.5	−0.6	−0.1	−1.1; 0.9	0.803

The analysis of sequence B (1^st^ Period: Filtered air – 2^nd^ Period: UFP) revealed no treatment differences between UFP and filtered air.

## 3 Discussion

We elucidated the pro-allergic effect of UFP on the airway inflammation in asthmatics using a two-treatment/two-period cross-over study design. We observed no significant effects of UFP on the allergic inflammation at 24 h after allergen challenge after UFP exposure compared to an exposure with filtered air within the associated treatment period. The failure to proof an acute UFP associated effect on the allergic reaction in asthmatics might be related to the sampling time point of 42 hours, which was based on previous data from an exposure study with identical UFP particles in a mouse model of allergic asthma [23]. However, our post hoc subgroup analysis revealed that subjects who were first exposed to UFP and allergen and 28 days later to filtered air and allergen showed a pronounced increase in inflammatory response, while patients treated with UFP and filtered air in the inverted order (sequence B) showed no significant effects. Moreover, the observed increased number of inflammatory cells in the BAL fluid, predominantly eosinophils and monocytes, were associated with an increased potential of BAL cells to generate oxygen radicals. Additionally, chemokines which are involved in the recruitment and differentiation of these inflammatory cells were found to be elevated.

The cross-over design of this study was based on the assumption from previous clinical exposures studies with UFP [13],[25], that a wash-out period of 28 days is sufficient to provide two independent treatment periods. However, in the second period of both treatment sequences a trend (p = 0.2) for an increased inflammatory response was seen. A potential interpretation is, that the experimental procedures (challenges with clean air/UFP, bronchoscopies and allergen challenges) of the first period had an influence on the second period. A carry-over effect of the experimental session can not be completely ruled out, although the formal carry-over analysis of all subjects was not significant (p = 0.2). Therefore, the negative findings from this study in the global analysis could be explained by the trend for a carryover effect between the exposures which may have biased the study towards the null. The fact however, that in the subgroup post hoc analysis the inflammatory effects during the 2^nd^ exposure period were statistically significant only for sequence B supports the speculation that the inhalation of UFP in the first period has an interaction on the allergen challenge in the second period. Therefore a wash-out period of 28 days between UFP exposure sessions is not sufficient, when interactions with allergen are considered. If such carry-over effects can not be completely ruled out and analysis of subgroups becomes necessary, small sample sizes in the subgroups (sequence A: n = 7; sequence B: n = 5) are a major limitation of the study. Therefore, the results have to be interpreted with caution and should be used to generate hypotheses for further investigations.

Despite the limitation of the study, the potential effects of the UFP on allergic inflammation 28 days after UFP exposure is an interesting speculation. One could speculate that UFP persist in the airways because they evade effective clearance mechanisms, e.g. the phagocytosis by alveolar macrophages and the mucociliar clearance, due to their ultrafine structure compared to larger particles [28] resulting in an inefficient clearance of inhaled nanoparticles [29],[30]. The persistence of UFP might maintain the activation of alveolar epithelial cells and macrophages via their high activity (i.e. oxygen radical production) leading to an enhanced inflammation when subjects are exposed to allergens later on. In order to define potential mechanisms, we have investigated a series of mediators in the BAL fluid 24 hours following the allergen challenge.

The increased concentration of the proinflammatory mediators IL-6, MCP-1 and TNF-alpha found in the BAL points to an involvement and preactivation of the innate immune compartment. As a possible mechanism it could be speculated that long living macrophages that have phagocytosed UFP particles at the first exposure might be primed for a enhanced reaction to a second stimulus, namely the allergen provocation. Therefore, the macrophages might release increased amounts of cytokines after activation by the T-cell dependent allergic inflammatory response. The secondary activation of the macrophages by the allergic cascade would also explain why the levels of Th2 cytokines were not elevated. However, the proinflammatory cytokines could contribute to the attraction of inflammatory cells into the airways. Similar data have been seen in the lung of an allergic mouse model following exposure to nano particles in the absence and presence of allergen [31].

No data are available how long UFP persist in human airways and how long a potential pro-allergic effect might be seen. In the mouse model of allergic asthma the study of Alessandrini et al. [23] has shown that the exposure to UFP prior to allergen challenge exerts strong adjuvant effect for several days, but data on long term effects are lacking. In our study only one single time point, namely 28 days after the UFP inhalation, is available for analysis. Furthermore, the influence of environmental particle exposure before the controlled challenges or during the 28 day recovery period was not controlled and can not be excluded. Therefore, further studies are necessary which investigate the potential longterm effect of UFPs on the allergic inflammation. These studies should be sufficiently powered and the potential carry-over effects should be avoided in a parallel group study design with repeated measurements. Ideally, the influence of external particle sources should be controlled or at least monitored throughout the study.

## 4 Conclusion

This study has expanded the limited available data about the causality of UFP and health effects, supporting the speculation that retained UFP might aggravate the response to allergens in asthma patients. However, this should be reconfirmed in further studies with an appropriate study design and sufficient number of subjects.

## 5 Methods

### 5.1 Study design

This randomized, double-blind, cross-over study was performed from October 2007 to March 2008 out of the pollen season at the Fraunhofer Institute for Toxicology and Experimental Medicine (ITEM) in Hannover, Germany. Allergic asthmatics were randomly assigned to two exposure subgroups starting with either ultrafine carbon particles followed by filtered air (sequence A), or filtered air followed by ultrafine carbon particles (sequence B). Each inhalation lasted for two hours in an environmental exposure chamber and was followed by a subsequent segmental allergen challenge during bronchoscopy 18 hours after the exposures with a second bronchoscopy 24 hours after allergen challenge to obtain the BAL cells. The consecutive inhalative exposures were separated by a recovery period of at least 28 days (see Figure 1). The study protocol (ClinicalTrails.gov Identifier: NCT00527462) was approved by the Ethics Committee of Hannover Medical School and was conducted in accordance to the Good Clinical Practice and the declaration of Helsinki. Written informed consent was provided by all subjects.

### 5.2 Randomization and blinding

The randomization code was generated using SAS software without blocking and stratification and provided to the physicists responsible for the generation of the inhalation atmosphere. All study subjects and clinical investigators were unaware about the exposure sequences.

### 5.3 Study subjects

Mild asthmatic subjects were recruited from a volunteer data base. All were non-smokers, allergic to grass pollen and had shown asthma symptoms in the previous grass pollen season (controlled by lung function and the Juniper Asthma control questionnaire©). They were able to abstain from treatment with corticosteroids, sodium cromoglycate, theophylline, or leukotriene modifiers and had no respiratory tract infections within 4 weeks before the start of the study procedures. During the study the subjects only used β2-agonists for relief of asthma symptoms, if needed. The airway hyperresponsiveness to methacholine was determined 7 days prior to the first exposure session (see Table 1).

### 5.4 Exposures to UFP and filtered air

The asthmatic subjects were exposed to a concentration of 64 μg/m^3^ UFP by oral breathing (with nose clip) for two hours in an environmental exposure chamber. During the exposures they performed an intermittent bicycle exercise with alternating 15 min periods of exercise and rest at an intensity adjusted to increase the minute ventilation to 20 L/min/m^2^. The dose of deposited particles was calculated to be equivalent to a 24 hours exposure in central urban regions in the western hemisphere. In urban air, the persistent insoluble part of the ultrafine particle concentration is primarily related to soot emitted from combustion processes. The soot concentration in Berlin, measured in the vicinity of major roads, ranges between 6.5 and 8 μg/m^3^ (annual mean values in the years 2000–2004) [32]. The artificially generated soot particles are similar in size and morphology to the soot particles freshly emitted by the road traffic. Assuming a daily 24 h exposure to 6.5 - 8 μg/m^3^ for people living in the vicinity of major roads would cause the same lung dose in volunteers exposed for 2 h to 78 – 96 μg/m^3^. Thus the selected concentration of 64 μg/m^3^ is slightly below but in the same range as the daily intake of people living close to major roads. To minimize interference from inhalation of high ambient particle concentrations (e.g. due to diesel (traffic) or cigarette smoke exposure) immediately prior to the controlled exposure sessions the subjects stayed overnight in the institute prior to the subsequent segmental allergen challenge.

The control exposure session also lasted for two hours and was performed under identical conditions as the UFP exposure except for using filtered air downstream of high efficiency particle filters (HEPA filter). Henceforth, we refer to an UFP exposure followed by a clean air exposure as sequence A and to the inverse order of exposure types as sequence B.

### 5.5 Generation and monitoring of UFP

In this study feshly generated carbon UFP were used for ambient urban UFP, which is dominated by motor engine combustion.

UFP were generated by electric spark discharge with a modified particle generator (Type GFG 1000, Palas, Germany) using highly purified elemental graphite electrodes in an argon atmosphere. The original spark discharge chamber was replaced by an inert ceramic chamber to avoid organic contamination of the carbon particles, as previously described [33],[34]. The carbon aerosol was generated at a production rate of 3 mg/h, electrically neutralized and transported at a volume flow rate of 3–8 L/min into the environmental exposure chamber (V = 15.5 m^3^) with walls of stainless steel. In order to achieve a concentration of 64 μg/m^3^ UFP, the chamber was ventilated with a dilution air stream (HEPA filtered) adjusted to 60 m^3^/h corresponding to an exchange rate of 4 per hour by a push pull ventilation system. The temperature and the relative humidity were in the range of 22–25°C and 40–60%, respectively. The air conditioning parameters were continuously measured by calibrated sensors.

The number-size distribution of the UFP in the exposure chamber was monitored continuously (once every 5 min) using an electrical mobility spectrometer (Model 3071/3025, TSI, USA). A condensation nucleus counter (Model 3010, TSI, USA) was used to measure 5-min average values of the total number concentration. Furthermore, cumulative filter samples were collected during the entire exposure period and the particle mass concentration was measured by gravimetrical analysis of the filter-deposited particle mass and the cumulative sampling volume.

### 5.6 Safety assessments during exposure session

Pulmonary function and blood pressure were measured every 30 minutes during the exposure with a hand-held asthma monitor (AM1®, CareFusion, Germany) and telemetric blood pressure meter, respectively. Additionally, the oxygen saturation and heart rate was monitored continuously via telemetric pulse oximetry and electrocardiogram (ECG), respectively.

### 5.7 Bronchoscopy and segmental allergen challenge

Eighteen hours after the exposure to UFP or filtered air, a first bronchoscopy was performed. Following baseline bronchoalveolar lavage (BAL) in the left lower lobe, allergen (grass pollen extract; ALK Scherax, Germany) in a volume of 10 ml saline solution as well as 10 ml saline solution as a control challenge were instilled in the contra lateral lung segments (Table 1), as previously described [35]. Twenty-four hours later, during a second bronchoscopy, BAL was performed in the allergen- and saline-challenged segments. The individual dose of allergen was calculated by a skin prick test performed with a 10-fold-dilution series of the allergen extract as previously described. The bronchoscopies were conducted under continued oxygen supplementation by certified pneumologists after premedication with midazolam according to a standard protocol following international recommendations for fiberoptic bronchoscopy (NHLBI workshop 1991) [36]. During the bronchoscopic procedure and the respective safety monitoring period thereafter, all subjects were continuously monitored with a three-lead ECG and pulse oximetry. Lung function measurement was performed prior to discharge of the patient. Subsequent home monitoring of the lung function was done by the subjects at home every two hours until bedtime and the next morning using a hand-held asthma monitor.

### 5.8 Processing and staining of BAL cells

BAL fluid samples were processed as previously described [37]. In brief, the BAL was centrifuged and the supernatant was stored at −80°C. The total nucleated cell count was determined using a Neubauer hemocytometer. Differential cell counts were obtained using Diff-Quick staining (Dade Behring Inc., Marburg, Germany). For the analysis of monocytes, flow cytometric BAL cell differentiation was performed using a Cytomics™ FC 500 cytometer (Beckman Coulter).

### 5.9 Oxygen radical generation of BAL cells

The production of reactive oxygen species by BAL cells was determined by measuring the chemiluminescence of lucigenin-loaded cells after stimulation as follows. BAL cells, at a concentration of 1 × 10^6^/ml, were incubated (30 minutes, 37°C) in HEPES-buffered RPMI with 5% AB serum containing 0.6 mM lucigenin (Sigma, Taufkirchen, Germany). Then, the response of the BAL cells to medium (control) or 10 μM PMA over a period of 30 minutes was determined. The data were expressed as integrated relative light units (RLU). This test determines the production of oxygen radicals by BAL cells upon non-specific mitogenic stimulation. The readout reflects the cellular composition of the BAL samples (e.g. eosinophils produce more oxygen radicals) and the activation status of the cells herein.

### 5.10 Biochemical analysis of BAL fluid

The concentration of cytokines and chemokines in the BAL fluid was determined with a BIO-PLEX Protein Array System (BIO-RAD Laboratories, USA) with premixed antibody-coated microsphere beads (Millipore, USA) according to the manufacturer’s recommendations.

### 5.11 Statistical analysis

The primary hypothesis of the two-treatment (the exposures)/two-period cross-over study was to show a significant difference of the allergen effect depending on whether a subject was pre-exposed to UFP or to filtered air. For each subject the allergen effect is represented by the difference of the measurements between the lung segment instilled with allergen and the segment instilled with saline. The primary endpoint was the number of total BAL cells. The key secondary endpoint was the number of eosinophils in the BAL fluid. For the primary analysis estimates and 95% confidence intervals for the allergen effect (UFP (allergen-saline) ↔ filtered air (allergen-saline)) were estimated in an analysis of variance with linear mixed effects consisting of the fixed factors of exposure, period, sequence, and subjects within sequence as a random factor.

Descriptive analyses include means and standard deviations for each sequence and period. Paired *t*-tests were reported to evaluate differences in the exposure effects between periods descriptively. Results were considered to be significant at a P value of <0.05. All data analyses were performed with SAS software, version 9.2.

Sample size estimation was based on the paired *t*-test. No prior information on the standard deviations was available, however it was assumed that an effect size of δ = ¾ = 0.75 would be of clinical relevance. Thus, a sample size of 16 patients would be sufficient to reach a power of 80% to detect relevant differences between exposition and control at a two-sided α-level of 5% (nQueryAdvisor 6.0).

## Abbreviations

BAL: Bronchoalveolar lavage

ECG: Electrocardiogram

FA: Filtered air

HEPA: High efficiency particulate air filter

PBS: Phosphate Buffered Saline System

PMA: Phorbol 12-myristate 13-acetate

UFP: Ultrafine particles

## Competing interests

The authors declare that they have no competing interests.

## Authors’ contributions

FS has made substantial contribution to study design (study protocol, performing the clinical trial), as well as data analysis and drafting the manuscript. CF and AK performed statistical analysis. DD contributed to data acquisition and data analysis. HW and WK established the UFP exposure. FA, HS, HB, MM, AB, JMH, and NK contributed to the study design. CW and OS contributed to drafting the manuscript. EK modified the particle generator for the UFP exposure. All authors read and approved the final manuscript.

## References

[B1] Understanding the health effects of ambient ultrafine particles 2013 2013 HEI Perspectives 3. Health Effects Institute, Boston, MA

[B2] Anderson JO Thundiyil JG Stolbach A Clearing the air: a review of the effects of particulate matter air pollution on human health J Med Toxicol 2012 8 166 175 10.1007/s13181-011-0203-1 22194192 PMC3550231

[B3] Pope CA III Dockery DW Health effects of fine particulate air pollution: lines that connect J Air Waste Manag Assoc 2006 56 709 742 10.1080/10473289.2006.10464485 16805397

[B4] Frampton MW Does inhalation of ultrafine particles cause pulmonary vascular effects in humans? Inhal Toxicol 2007 19 Suppl 1 75 79 10.1080/08958370701495071 17886054

[B5] Gauderman WJ Air pollution and children–an unhealthy mix N Engl J Med 2006 355 78 79 10.1056/NEJMe068096 16823000

[B6] Knol AB de Hartog JJ Boogaard H Slottje P van der Sluijs JP Lebret E Cassee FR Wardekker JA Ayres JG Borm PJ Brunekreef B Donaldson K Forastiere F Holgate ST Kreyling WG Nemery B Pekkanen J Stone V Wichmann HE Hoek G Expert elicitation on ultrafine particles: likelihood of health effects and causal pathways Part Fibre Toxicol 2009 6 19 10.1186/1743-8977-6-19 19630955 PMC2731037

[B7] Lippmann M Health effects of airborne particulate matter N Engl J Med 2007 357 2395 2397 10.1056/NEJMe0706955 18057343

[B8] Oberdorster G Gelein RM Ferin J Weiss B Association of particulate air pollution and acute mortality: involvement of ultrafine particles? Inhal Toxicol 1995 7 111 124 10.3109/08958379509014275 11541043

[B9] Alessandrini F Beck-Speier I Krappmann D Weichenmeier I Takenaka S Karg E Kloo B Schulz H Jakob T Mempel M Behrendt H Role of oxidative stress in ultrafine particle-induced exacerbation of allergic lung inflammation Am J Respir Crit Care Med 2009 179 984 991 10.1164/rccm.200807-1061OC 19264975

[B10] Geiser M Kreyling WG Deposition and biokinetics of inhaled nanoparticles Part Fibre Toxicol 2010 7 2 10.1186/1743-8977-7-2 20205860 PMC2826283

[B11] Li N Sioutas C Cho A Schmitz D Misra C Sempf J Wang M Oberley T Froines J Nel A Ultrafine particulate pollutants induce oxidative stress and mitochondrial damage Environ Health Perspect 2003 111 455 460 10.1289/ehp.6000 12676598 PMC1241427

[B12] Atkinson RW Anderson HR Sunyer J Ayres J Baccini M Vonk JM Boumghar A Forastiere F Forsberg B Touloumi G Schwartz J Katsouyanni K Acute effects of particulate air pollution on respiratory admissions: results from APHEA 2 project. Air pollution and health: a European approach Am J Respir Crit Care Med 2001 164 1860 1866 10.1164/ajrccm.164.10.2010138 11734437

[B13] Chalupa DC Morrow PE Oberdorster G Utell MJ Frampton MW Ultrafine particle deposition in subjects with asthma Environ Health Perspect 2004 112 879 882 10.1289/ehp.6851 15175176 PMC1242016

[B14] Strickland MJ Darrow LA Klein M Flanders WD Sarnat JA Waller LA Sarnat SE Mulholland JA Tolbert PE Short-term associations between ambient air pollutants and pediatric asthma emergency department visits Am J Respir Crit Care Med 2010 182 307 316 10.1164/rccm.200908-1201OC 20378732 PMC2921597

[B15] Tolbert PE Mulholland JA MacIntosh DL Xu F Daniels D Devine OJ Carlin BP Klein M Dorley J Butler AJ Nordenberg DF Frumkin H Ryan PB White MC Air quality and pediatric emergency room visits for asthma in Atlanta, Georgia, USA Am J Epidemiol 2000 151 798 810 10.1093/oxfordjournals.aje.a010280 10965977

[B16] Zhang JJ McCreanor JE Cullinan P Chung KF Ohman-Strickland P Han IK Järup L Nieuwenhuijsen MJ Health effects of real-world exposure to diesel exhaust in persons with asthma Res Rep Health Eff Inst 2009 ᅟ 5 109 19449765

[B17] McCreanor J Cullinan P Nieuwenhuijsen MJ Stewart-Evans J Malliarou E Jarup L Harrington R Svartengren M Han IK Ohman-Strickland P Chung KF Zhang J Respiratory effects of exposure to diesel traffic in persons with asthma N Engl J Med 2007 357 2348 2358 10.1056/NEJMoa071535 18057337

[B18] Kelly FJ Fussell JC Air pollution and airway disease Clin Exp Allergy 2011 41 1059 1071 10.1111/j.1365-2222.2011.03776.x 21623970

[B19] Pope CA III Epidemiology of fine particulate air pollution and human health: biologic mechanisms and who's at risk? Environ Health Perspect 2000 108 Suppl 4 713 723 10.2307/3454408 10931790 PMC1637679

[B20] de Haar C Hassing I Bol M Bleumink R Pieters R Ultrafine but not fine particulate matter causes airway inflammation and allergic airway sensitization to co-administered antigen in mice Clin Exp Allergy 2006 36 1469 1479 10.1111/j.1365-2222.2006.02586.x 17083358

[B21] Granum B Gaarder PI Groeng E Leikvold R Namork E Lovik M Fine particles of widely different composition have an adjuvant effect on the production of allergen-specific antibodies Toxicol Lett 2001 118 171 181 10.1016/S0378-4274(00)00292-7 11137324

[B22] Kang X Li N Wang M Boontheung P Sioutas C Harkema JR Bramble LA Nel AE Loo JA Adjuvant effects of ambient particulate matter monitored by proteomics of bronchoalveolar lavage fluid Proteomics 2010 10 520 531 10.1002/pmic.200900573 20029843 PMC3021977

[B23] Alessandrini F Schulz H Takenaka S Lentner B Karg E Behrendt H Jakob T Effects of ultrafine carbon particle inhalation on allergic inflammation of the lung J Allergy Clin Immunol 2006 117 824 830 10.1016/j.jaci.2005.11.046 16630940

[B24] Daigle CC Chalupa DC Gibb FR Morrow PE Oberdörster G Utell MJ Frampton MW Ultrafine particle deposition in humans during rest and exercise Inhal Toxicol 2003 15 539 552 10.1080/08958370304468 12692730

[B25] Frampton MW Utell MJ Zareba W Oberdörster G Cox C Huang LS Morrow PE Lee FE Chalupa D Frasier LM Speers DM Stewart J Effects of exposure to ultrafine carbon particles in healthy subjects and subjects with asthma Res Rep Health Eff Inst 2004 ᅟ 1 47 15768531

[B26] Frampton MW Stewart JC Oberdörster G Morrow PE Chalupa D Pietropaoli AP Frasier LM Speers DM Cox C Huang LS Utell MJ Inhalation of ultrafine particles alters blood leukocyte expression of adhesion molecules in humans Environ Health Perspect 2006 114 51 58 10.1289/ehp.7962 16393658 PMC1332656

[B27] Krug N Teran LM Redington AE Gratziou C Montefort S Polosa R Brewster H Howarth PH Holgate ST Frew AJ Carroll MP Safety aspects of local endobronchial allergen challenge in asthmatic patients Am J Respir Crit Care Med 1996 153 1391 1397 10.1164/ajrccm.153.4.8616571 8616571

[B28] Kreyling WG Semmler-Behnke M Takenaka S Moller W Differences in the biokinetics of inhaled nano- versus micrometer-sized particles Acc Chem Res 2013 46 714 722 10.1021/ar300043r 22980029 PMC3556515

[B29] Oberdörster G Finkelstein JN Johnston C Gelein R Cox C Baggs R Elder AC Acute pulmonary effects of ultrafine particles in rats and mice Res Rep Health Eff Inst 2000 ᅟ 5 74 11205815

[B30] Oberdorster G Oberdorster E Oberdorster J Nanotoxicology: an emerging discipline evolving from studies of ultrafine particles Environ Health Perspect 2005 113 823 839 10.1289/ehp.7339 16002369 PMC1257642

[B31] Inoue K Takano H Yanagisawa R Ichinose T Sakurai M Yoshikawa T Effects of nano particles on cytokine expression in murine lung in the absence or presence of allergen Arch Toxicol 2006 80 614 619 10.1007/s00204-006-0075-3 16482471

[B32] Lutz M Lehming B Breitenkamp M Luftreinhalte- und Aktionsplan Berlin 2005-2010 2005 Senatsverwaltung für Stadtentwicklung, Berlin

[B33] Matuschek G Karg E Schroppel A Schulz H Schmid O Chemical investigation of eight different types of carbonaceous particles using thermoanalytical techniques Environ Sci Technol 2007 41 8406 8411 10.1021/es062660v 18200871

[B34] Roth C Ferron G Karg E Lentner B Schuhmann G Takenaka S Heyder J Generation of ultrafine particles by spark discharging Aerosol Sci Technol 2004 38 228 235 10.1080/02786820490247632

[B35] Schaumann F Müller M Braun A Luettig B Peden DB Hohlfeld JM Krug N Endotoxin augments myeloid dendritic cell influx into the airways in patients with allergic asthma Am J Respir Crit Care Med 2008 177 1307 1313 10.1164/rccm.200706-870OC 18388357 PMC2427055

[B36] Guidelines for fiberoptic bronchoscopy in adults Am Rev Respir Dis 1987 136 1066 10.1164/ajrccm/136.4.1066 3662229

[B37] Schaumann F Borm PJ Herbrich A Knoch J Pitz M Schins RP Luettig B Hohlfeld JM Heinrich J Krug N Metal-rich ambient particles (particulate matter 2.5) cause airway inflammation in healthy subjects Am J Respir Crit Care Med 2004 170 898 903 10.1164/rccm.200403-423OC 15229099

